# Harnessing the PRECISE network as a platform to strengthen global capacity for maternal and child health research in sub-Saharan Africa

**DOI:** 10.1186/s12978-020-0876-5

**Published:** 2020-04-30

**Authors:** Meriel Flint-O’Kane, Peter von Dadelszen, Prestige Tatenda Makanga, Esperança Sevene, Anna Roca, Peter Dukes, Saba Hinrichs-Krapels, Rachel Craik, Laura A. Magee, Marleen Temmerman, The PRECISE Network, Umberto D’Alessandro, Anna Roca, Hawanatu Jah, Ofordile Oguchukwu, Andrew Prentice, Melisa Martinez-Alvarez, Brahima Diallo, Adbul Sesey, Kodou Lette, Alpha Bah, Chilel Sanyang, Marleen Temmerman, Angela Koech Etyang, Peris Musitia, Mary Amondi, David Chege, Patricia Okiro, Geoffrey Omuse, Sikolia Wanyonyi, Esperança Sevene, Paulo Chin, Corssino Tchavana, Salesio Macuacua, Anifa Vala, Helena Boene, Lazaro Quimice, Sonia Maculuve, Eusebio Macete, Inacio Mandomando, Carla Carillho, Peter von Dadelszen, Laura A. Magee, Meriel Flint-O’Kane, Rachel Craik, Amber Strang, Marina Daniele, Donna Russell, Tatenda Makanga, Liberty Makacha, Yolisa Dube, Newton Nyapwere, Aris Papageorgiou, Alison Noble, Hannah Blencowe, Veronique Filippi, Joy Lawn, Matt Silver, Matthew Chico, Judith Cartwright, Guy Whitley, Sanjeev Krishna, Marianne Vidler, Jing Larry Li, Jeff Bone, Mai-Lei Maggie Woo Kinshella, Beth A. Payne, Domena Tu, Warancha Tumtaweetikul, William Stones

**Affiliations:** 1 Department of Women and Children’s Health, School of Life Course Science, Faculty of Life Sciences and Medicine, King’s College London, London, UK; 2 Faculty of Public Health and Policy, London School of Hygiene and Tropical Medicine, London, UK; 3 Midlands State University, Gweru, Zimbabwe; 4 Department of Physiological Science, Clinical - Pharmacology, Faculty of Medicine, Universidade Eduardo Mondlane, Maputo, Mozambique; 5 Centro de Investigação em Saúde de Manhiça, Manhiça, Maputo Province Mozambique; 6 Medical Research Council Unit (The Gambia) at the London School of Hygiene and Tropical Medicine, Fajara, The Gambia; 7 Africa Research Excellence Fund, Fajara, The Gambia UK; 8 Africa Research Excellence Fund, London, UK; 9 The Policy Institute, King’s College London, London, UK; 10 Nuffield Department of Women’s and Reproductive Health, University of Oxford, Oxford, UK; 11 Centre of Excellence in Women and Child Health, East Africa, Aga Khan University, Nairobi, Kenya

**Keywords:** Pregnancy, Capacity building, Global Health, Leadership, Africa south of the Sahara, United Kingdom

## Abstract

It is widely acknowledged across the global health sector that research programmes need to be designed and implemented in a way that maximise opportunities for strengthening local capacity. This paper examines how the United Kingdom Research and Innovation (UKRI) Grand Challenges Research Fund (GCRF) funded PRECISE (PREgnancy Care Integrating translational Science, Everywhere) Network has been established as a platform to strengthen global capacity for research focused on the improvement of maternal, fetal and newborn health in sub-Saharan Africa.

Best practice principles outlined in an ESSENCE on Health Research report have been considered in relation to the PRECISE Network capacity-building activities described in this paper. These activities are described at the individual, programmatic and institutional levels, and successes, challenges and recommendations for future work are outlined.

The paper concludes that the PRECISE leadership have an opportunity to review and refresh activity plans for capacity building at this stage in the project to build on achievements to date.

## Background

Effective, innovative approaches and solutions to global health challenges are built on evidence and best practices, and knowledge gathering for this purpose benefits from collaboration between researchers from different disciplines and regions. A research partnership bringing together experts from less-developed countries, working collaboratively with colleagues from the global north, needs to embrace capacity building in research to create an architecture that will advance science that is relevant to low-and-middle-income countries.

When investing Official Development Assistance (ODA) budgets, UK research funders often state criteria for explicitly-described outputs relating to developing research leadership and capacity through global health projects and programmes [1–5]. Embedding these activity packages alongside and within research plans is seen as a mechanism for increasing the sustainability of investments through strengthening the infrastructure (institutional capacity building), and human capital (building capacity through investing in individuals) of the research system in the UK and for partners in less-developed countries. Such investments are aimed not only at increasing the capacity for research production but also for research dissemination and uptake to maximise impact [5]. The necessity of including capacity-strengthening work packages in global health research is not unique to the UK. National and international actors across the global health research environment recognise and endorse these activities as pre-requisites for effective and sustainable development in this field. This is demonstrated by the inclusion of capacity building themes across WHO programmes [6–8].

The ESSENCE on Health Research initiative released a good practice document for capacity strengthening in 2014, entitled *Seven principles for strengthening research capacity in low- and middle-income countries: simple ideas in a complex world* [9]. The principles proposed are:

1. Network, collaborate, communicate and share experiences
2. Understand the local context and accurately evaluate existing research capacity
3. Ensure local ownership and secure active support
4. Build in monitoring, evaluation and learning from the start
5. Establish robust research governance and support structures, and promote effective leadership
6. Embed strong support, supervision and mentorship structures
7. Think long-term, be flexible and plan for continuity

This paper explores these themes in the context of the United Kingdom Research and Innovation (UKRI) Grand Challenges Research Fund (GCRF) Growing Research Capacity (GROW) Scheme-funded PRECISE (PREgnancy Care Integrating translational Science, Everywhere) Network. The authors review how this international research programme has sought to incorporate capacity building at individual, programmatic and institutional levels according to these principles.

## Introduction

**Fig. 1:**
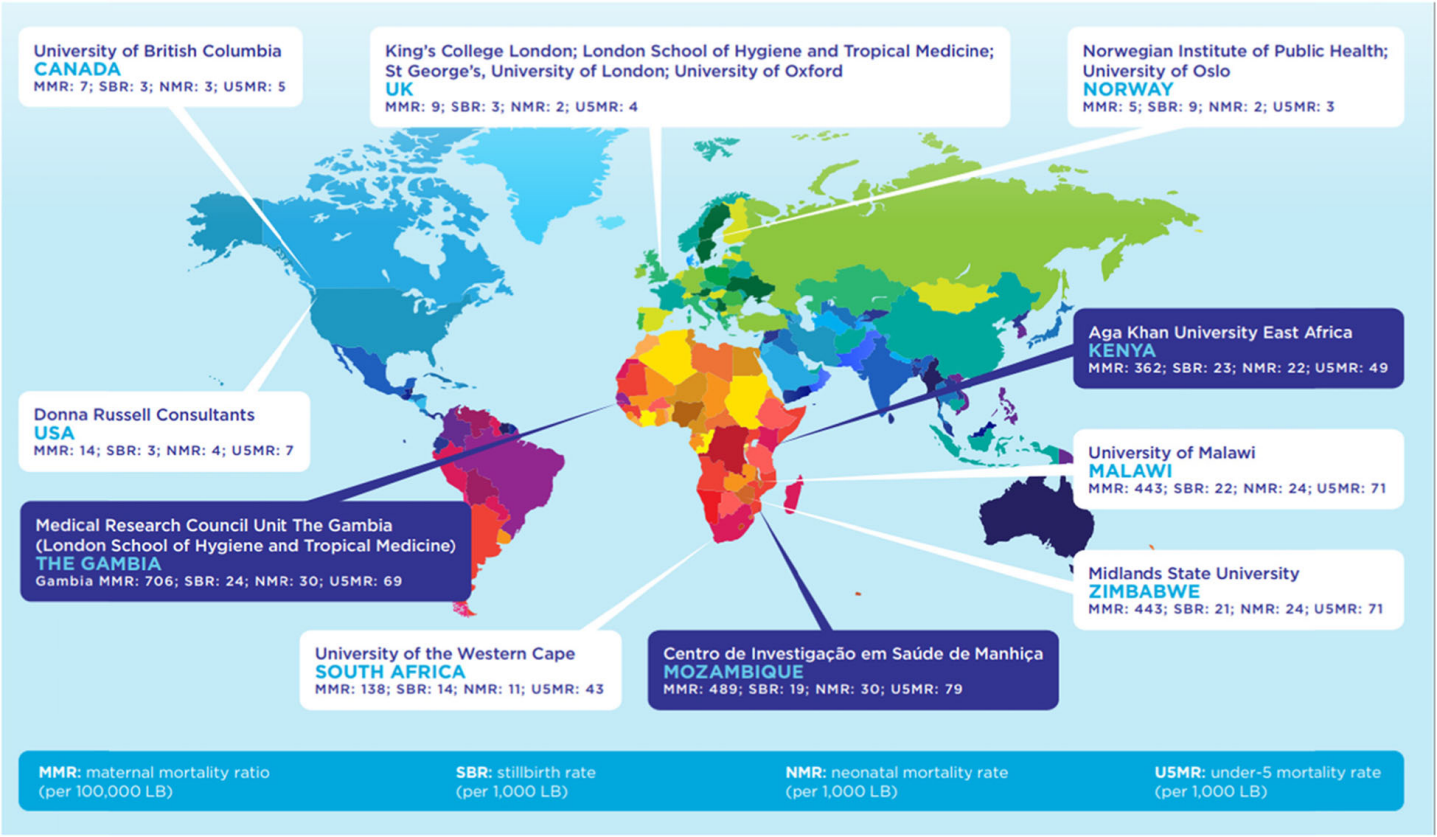
Map of PRECISE partners

The PRECISE Network (Fig. 1) is funded by the UKRI GCRF GROW call which had the specific aim of developing “research capacity around the globe and to strengthen and broaden skills and expertise to address specific challenges of developing regions and countries” [1]. As is shown by the first four papers in this BMC Reproductive Health Supplement, An Introduction to The PRECISE Network [10–13], this programme of work aims to achieve capacity building through the development of an international team that will set-up a pregnancy biobank and develop a holistic data set to enable deep phenotyping of factors affecting placental disease in three sub-Saharan African settings. This is summarised in the first of the four PRECISE Network aims, “Build individual and institutional research capacity across Africa and the UK through a shared pregnancy research programme of work” [14].

The Network has been established as a collaboration between 13 research institutions and is hosted at King’s College London in the UK.

This paper describes the PRECISE Network plans for developing organisational and human capacity across individual, programme and institutional levels, and summarise the achievements and challenges to date. Recommendations for next steps to achieve Aim 1 of The Network are described.

### Building global research capacity through career support for individuals

Harnessing and developing the skills of colleagues involved in the Network across all role types and career stages is an important opportunity for PRECISE. Although utilising the scale and academic infrastructure of the Network to support individuals is an ‘obvious’ mechanism through which to build human capacity across the global women’s and children’s health research architecture, with the competing demands of global health programme delivery it is known to be very challenging to find the resources (both time and financial) to fully maximise the opportunity for large global networks to effectively build research capacity. The senior investigator and management team in PRECISE have prioritised investment in career support for talented individuals across the research and fieldwork teams in two main ways:

1. By partnering with the Africa Research Excellence Fund (AREF) [15] to deliver a “soft skills” training programme ‘Towards Leadership’ for the rising star researchers (identified through an application and selection process) involved in PRECISE who are based in Africa (https://precisenetwork.org/research_themes/research-leadership-development/).

2. By working with The Global Health Network [16] to establish a platform on which individuals working on PRECISE can access further training and enhance their skills and CV’s through the opportunities this Network provides.

The Towards Leadership programme aims to ensure strong ‘support, supervision and mentorship structures’ within PRECISE (ESSENCE principle 6), and was launched in partnership with two other GCRF GROW awardees, CAN (the Crick Africa Network), hosted at The Crick Institute, and RECAP (Research capacity building and knowledge generation to support preparedness and response to humanitarian crises and epidemics), hosted at LSHTM.

The objectives of the Towards Leadership programme are:

- To enhance the soft skills of African “fellows” (the researchers on the Programme) that are essential to managing and leading global health research
- To empower fellows to develop and manage fulfilling careers
- To add value to research training, experience and opportunities provided by CAN, RECAP and PRECISE.

The programme is grounded in theory and good practice, while emphasising practical, experiential learning. The curriculum is based on that of AREF’s *Excell* programme [17], itself based on needs assessment and in alignment with the Vitae Researcher Development Framework [18].

Towards Leadership takes place over 10 months, with three face-to-face workshops in which Fellows from across PRECISE, CAN and RECAP come together with AREF facilitators to learn, share experiences and grow together as a cohort of future African research leaders. Fellows from a variety of disciplinary backgrounds spanning discovery science to medical anthropology were included in the cohort, so that they can share experiences about the challenges across the various academic disciplines and gain insights for future collaborations. Additionally, the cohort includes academics from East, West and Southern sub-Saharan Africa to build a cross-continent network of peers who can work and develop together, and/or provide mentorship and support for one another throughout their careers. AREF have found that a key challenge is how the growing confidence and newly-acquired skills can be embedded and sustained not only in Fellows’ own personal practice but also in the capacity strengthening strategy of their home institutions. Monitoring and evaluation plans to track key indicators that will enable an understanding of the success of this Programme, are in development between AREF and the Programme sponsors; Annex 1 which describes a Theory of Change workshop conducted to initiate the monitoring and evaluation process, in alignment with point 4 of the ESSENCE framework.

The shared vision for this leadership programme is that it will run for the remaining two years of GROW funding in 2020 and 2021, with fellows from across the three cohorts and in partnership with AREF. The partners are discussing evaluation plans for Year 1 of the programme and have conducted a Theory of Change workshop led by The Policy Institute, King’s College London, with 13 participants from across the partnership including senior research and management teams, AREF staff and programme Fellows. Learning from the Year 1 evaluation and Theory of Change development process (Appendix) will inform programme plans for future years. Sustainability of the initiative beyond this time will be dependent of available funding for capacity building programmes in the global health research environment.

To enhance learning and development across fieldwork teams PRECISE has partnered with The Global Health Network (TGHN) (https://precisecommunity.tghn.org/) which provides free e-learning modules. Through TGHN PRECISE has offered all staff access to the following professional development courses: Introduction to Clinical Research, Good Clinical Practice, Introduction to Informed Consent, Introduction to Data Management for Clinical Research Studies and an Introduction to Good Clinical Laboratory Practice. Certificates are available upon completion of each module and acquisition of new knowledge and skills can be tracked on the TGHN Professional Development Scheme which supports staff with career development. Partnering with TGHN helps provide the training and support required to ensure strong local ownership of programme implementation which is core to the PRECISE ethos and ESSENCE principle 3.

### Programme level capacity building in global research activities

A major legacy of the PRECISE programme will be the establishment of combined pregnancy multidimensional databases and biorepositories in Kenya, The Gambia and Mozambique [11, 12]. The combination of biological samples and extensive phenotypic data will enable new research into the biological pathways and co-exposures that cause, confound and affect the impact of placental disease (i.e., pre-eclampsia, fetal growth restriction, preterm birth and stillbirth) in sub-Saharan Africa. These data and samples will be a unique resource for the development of research activity at AKU, CISM, MRCG and other African institutions. PRECISE has ensured that these resources will support African science by confirming that all data and samples will be owned by the in-country academic institutions. Sample governance for PRECISE will be managed by a Data and Sample Access Committee with senior representation from all participating countries to discuss prioritisation and process for data and sample sharing across and beyond the consortium for future research (ESSENCE principles 5 and 7).

All equipment procured for PRECISE across clinical and biobanking activity, will remain at the African partner institutions to support their programmes of research activity. As a fundamental component of establishing the in-country biorepositories, we are developing local biorepository management and technical expertise. In the community setting, local staff are being trained to follow standard operating procedures for the collection, processing, and storage, and transport of biological specimens. PRECISE data and laboratory managers from AKU, MRCG and CISM attended a training week in Cape Town in April 2018 to learn about the BAOBAB Laboratory Information Management System (LIMS) [19] that PRECISE will use and is the preferred LIMS for B3Africa [20].

In addition to the ‘hardware’ of data and samples that PRECISE will deliver, it is important to note the ‘softer’ outputs the Network is facilitating through endeavours such as new institutional partnerships and opportunities for collaboration both across countries and academic disciplines. There is a strong and demonstrated commitment to ensuring PRECISE is open to the input and ideas of a broad range of colleagues to harness the immense collective expertise of the team to deliver this programme of work. Since the award started in October 2017 there have been two PRECISE Annual Meetings, both held in sub-Saharan Africa (Nairobi and Johannesburg) with over 80 attendees including professors, PhD students, research assistants, clinicians, laboratorial technicians, social scientists, database developers, programme managers, study co-ordinators, at all career stages. The aims of these meetings are well summarised by point 1 of the ESSENCE Framework; ‘Network, collaborate, communicate and share experiences’, as the primary purpose has been to bring together this broadly based groups of researchers from different academic and cultural backgrounds to co-develop plans for the scientific vision and implementation activities of PRECISE.

It is hoped that the process of learning together through the challenging set-up phase of PRECISE has laid the foundations for strong international and interdisciplinary relationships. These will be sustained through ancillary projects that utilise PRECISE data, samples and networks to further knowledge and understanding of placental disease. Many funding applications have already been submitted by PRECISE sub-groups for ancillary research questions that will build on PRECISE outputs and infrastructure (see Table 1). The long-term vison for continuity of PRECISE activities aligns with ESSENCE principle 7 that emphasises the importance of sustainability in working to strengthen capacity for research in LMIC’s.

**Table 1:** Ancillary funding applications building on the PRECISE platform

Project title	Lead Institution	Kenya	The Gambia	Mozambique	Zimbabwe
Geo-PriMACH: Geographical influences on perinatal, maternal and child health outcomes	MSU, Zimbabwe				X
GRIP (Group Inter-Pregnancy care) – an opportunity to improve health trajectories	KCL, UK	X			
MICA: PRECISE Discovery Science Partnership: working to reduce the burden of placental and related pregnancy disorders in sub-Saharan Africa	KCL, UK	X	X	X	
PRECISE-DYAD: linking maternal and infant health trajectories in sub-Saharan Africa	KCL, UK	X	X		
Measuring Respectful Maternity Care for the mother-baby dyad in Sub-Saharan Africa	KCL, UK	X	X	X	
PRECISE-CLEAR: Cardiovascular health and Links to the Environment in African Regions	KCL, UK	X	X	X	
Integrating mental health into maternal care in The Gambia, Kenya and Mozambique: situation analysis and contextual mapping	KCL, UK	X	X	X	
E-Registries in MNCH	KCL, UK	X	X	X	
Stillbirths – more than a mother’s loss; reducing stillbirth, improving care and changing attitudes around stillbirth in the Kenyan context	AKU, Kenya	X			
PIERS On the Move: from clinical trial tool to platform for clinical impact at scale	KCL, UK	X	X	X	

### Institutional capacity strengthening

#### Research governance (ESSENCE principle 5)

A strong common framework for research governance is an essential starting requirement to ensure all partners are operating according to shared principles and standards. During the programme set-up phase, it has been vital to establish strong working relationships with the research management and support functions at all partner institutions. This has enabled the completion of contract signing and movement of funds, due diligence processes to satisfy funder requirements, ethical approval for proposed research activities and many other activities essential for effective research governance. Mutual recognition of, and respect for, the different processes and pressures on participating institutions depending on their geographical location, institutional histories, role in the project, have enabled a collaborative environment in which all contracts, approvals, reports and audit requirements have been satisfied to date. It is testament to the expertise and resilience of the research support functions at all partner institutions that this has been possible. There has been shared learning across all parties that has inherently developed institutional capacity for international collaboration and managing programmes of this scale. Going forward, all PRECISE members will be required to report outcomes on Research Fish as a requirement of the funder, UKRI. Use of this internationally recognised evaluation platform as a tool for recording and demonstrating research output could be useful for ongoing research impact assessment. Future opportunities that could be explored by PRECISE include consideration of adopting the Good Financial Grants Practice (GFGP) platform [21] for long term assurance of internationally recognised best finance practice.

#### Advocacy for research

Feedback from across the Africa-based partners highlights lack of institutional support for research as a systemic barrier to the growth of academic research programmes regionally. This can make it extremely challenging for academics to build research careers at African institutions as incentives are structured to encourage undergraduate teaching over developing research portfolios. PRECISE aims to support the profile and image of research in the partner institutions through bringing funds but also recognition of the international communities’ commitment and interest in collaborating with African academics. More specifically, PRECISE hopes to highlight women’s and children’s health research as an area of focus for collaborators and funders globally. The maturity of existing maternal-fetal-newborn health research programmes varies across PRECISE partners in Africa; AKU has an established programme of research in this area whereas CISM and MRCG have traditionally had a greater focus on infectious disease research. This presents an opportunity to support the development of maternal-fetal-newborn health research portfolios according to the future research strategies and interests of partners. Recommendations for future planning by the consortium are noted in the ‘learning and recommendations’ section of this paper.

## Challenges in optimising PRECISE as a platform to strengthen global capacity for MCH research

This discussion will focus on two overarching issues both at the programme level and in the wider global health research environment; prioritisation and timelines.

Balancing priorities between setting up the ambitious infrastructure and research activities within PRECISE (see papers 1,2,3 and 4 of this supplement), and the need to mindfully utilise these platforms as opportunities to develop capacity has been a challenge for PRECISE. Much of the programme-level and institutional capacity building that has been achieved has been implicit within the process of building a global team to develop an appropriate database and establish the biobanking platforms ready for data and sample collection. This is a significant programmatic achievement that will support institutional capacity for partners; however, an important priority is to ensure that there is support for individuals to develop skills for their professional development. Given the immense demands on the time of senior investigators across their academic and administrative commitments, their scope to take on significant mentorship roles is limited. Incentive structures in academia are heavily structured towards outputs such as publications with high impact factors, and additional research funding secured. These incentives do not align with the need for time to be spent with junior colleagues to provide guidance and career support. Though senior staff are often extremely generous with their limited time, the globally-recognised structural barriers (as described above) within academia to effective capacity building through mentorship should be acknowledged. PRECISE has sought to augment the career support offered through mentorship by partnering with AREF and TGHN as described above to embed professional development within PRECISE Network activities. The combined cost of running these activities through the life of the project is less than £80,000 (assuming five PRECISE AREF fellows/year for 3 years), which is equal to 1% of the total budget and represents excellent value for money, relative to the scale of spending in PRECISE, as a mechanism for strengthening the legacy of this Network through supporting individuals.

Time pressure is a challenge for capacity building at both the micro and macro level. Within PRECISE, the need to deliver on research outcomes has been essential to ensuring programme completion within the four-year funding envelope. This has resulted in reduced time available for mentoring as described above and for planning and delivery of capacity building work-streams. At the ‘macro’ level, the extent to which a four-year programme can promise to effectively build and sustain research capacity within the global academic architecture is limited. Whilst every avenue to build on and link with existing strategies, platforms and programmes is being explored to secure the legacy of PRECISE, substantial challenges to continuation of the activities initiated within this programme are inevitable with a four-year funding envelope. It is understood that, especially when dealing with government donors, funding cycles are tied to wider pressures such as spending reviews and leadership changes, making longer term financial planning problematic. These broader contextual issues are important to recognise when evaluating the potential impact of capacity building activities and programmes.

### Learning and recommendations

As the set-up of programme infrastructure is complete there is now time to reflect on achievements in relation to capacity building as outlined in this paper, and plan for further strengthening of these activities to deliver sustainable outputs. It is noted that the body of work described here demonstrates clear alignment of the activities to date with six of the seven ESSENCE principles, however point 2 of the framework; ‘understand the local context and accurately evaluate existing research capacity’ is not explicitly discussed. The primary recommendation is to address this by spending time with partners across the consortium to work through a planning framework, such as the ‘5 step approach’ designed by Liverpool School for Tropical Medicine’s Centre for Capacity Research [22], which would provide a useful tool to inform this process. Due to the focus on collaborative development of the research protocol and scientific/clinical operating procedures in the first 18 months of PRECISE, it has not been possible to prioritise this activity previously. It will be important to milestone future plans according to funding availability as the PRECISE Network award ends in December 2021. Short-to-medium term outputs can be achieved within this timeframe however longer-term plans to deliver on agreed outcomes and desired impacts will require continued funding which PRECISE collaborators are working to secure across both programme and country levels (as demonstrated in Table 1).

## Conclusion

This paper has described mechanisms across the individual, programme and institutional levels through which PRECISE is working to build global capacity and expertise for maternal-fetal-newborn health research according to the ESSENCE principles. Across all three levels, the project set-up and implementation has been designed to support academic advancement and growth of research capacity and autonomy in Africa. The programme has developed activities that explicitly address PRECISE Aim 1, with a focus on training and development opportunities relevant to colleagues across the Network. Time pressure is highlighted as the primary risk to building effective individual and institutional research capacity across Africa and the UK through the Network. Understanding this as a challenge both within the programme but also as a threat to sustainability of outcomes in the wider context, will be essential for the longer-term evaluation of all GROW awards. In conclusion, the achievements and challenges to date are noted and the opportunity for PRECISE leadership to further utilise established tools and frameworks [9, 22] to develop phase 2 activity plans for capacity building that are rooted in local context and needs assessments is highlighted as an opportunity to build on successes to date.

## Figures and Tables

**Fig. 2:**
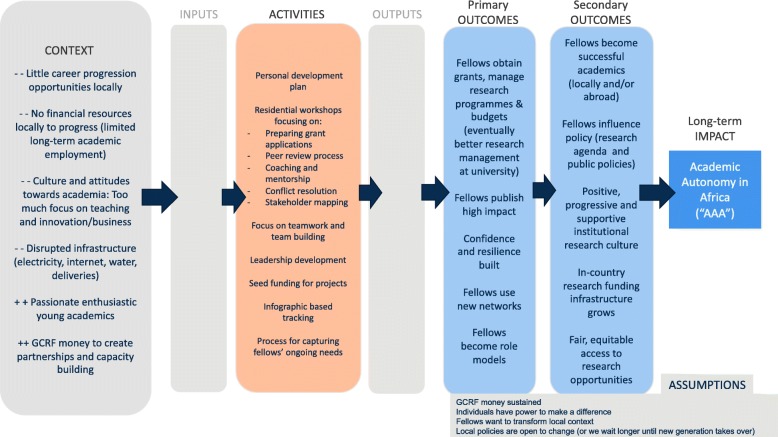
Draft theory of change model for Towards Leadership Programme

## Appendix

### Overview of results from a Theory of Change workshop for the Towards Leadership programme

This workshop focussed on identifying the needs of the local context (researchers in African institutions), the ultimate vision and impact desired, and the primary (or immediate) and secondary outcomes (intermediary) leading to that long-term impact.

The resulting logic model (Fig. 2) presents a summary of the discussions during the workshop. The goal of the workshop was to test assumptions about what the Programme can achieve, and ensure unity of vision with regards to its overarching ambition, with the experiences so far in the first few months of delivering the pilot programme in the first cohort of 2019. It was not yet intended to present detail of the activities of the Programme and any indicators for measuring success (which is why Inputs and Outputs are omitted from this initial draft). Having a variety of people in the room (from Programme managers to Fellows benefitting from the Programme) helped us achieve this goal and was a necessary step before monitoring and evaluation strategies were established. Oc.

The aim is to continue to update this model with the Programme team, so that it can be used for either monitoring and evaluation internally, and/or for communicating results of the Programme over time. Notes on the diagram are provided below.

#### Notes

Context: Discussions around context were focussed on understanding the Programme users’ needs, to re-focus on the purpose for running this Programme. Items with a “-” are considered challenges; items with a “+ +” are considered assets/strengths.

Inputs: These are the resources that have been put into running this Programme (e.g. funding, know-how, employees, existence of partnerships, curriculum availability, etc). These were not covered during the workshop, but will be reviewed and added as this initial model is developed for evaluation purposes.

Activities: These are the activities conducted to run the Programme, and were only roughly covered on the day. It is intended that some of these will be renewed and developed as the plans and strategies for the future of the Programme progress, in light of the renewed focus on the outcomes and ultimate impact the Programme wishes to achieve.

Outputs: These will be the indicators that demonstrate whether the activities are being conducted adequately. These were not covered on the day but it is intended that such outputs will be developed (along with indicators for success) for monitoring and evaluation purposes. Examples include number of Fellows going through the Programme; rate of participation in online courses; number of grants applied for/won by Fellows.

Primary and Secondary outcomes: We have detailed primary and secondary outcomes to demonstrate that there is some degree of chronology to these, but note that this is often not a linear process, as some of these outcomes have feedback loops and may overlap.

Impact: We spent a good portion of the workshop discussing the ultimate vision and long-term impact for the Programme. This helps the Programme team keep one clear goal in mind which unifies thoughts and activities. It is anticipated that the exact wording for this vision may have to be tailored for different audiences as appropriate.

Assumptions: The outlined pathway from primary to secondary outcomes, and to impact, makes many assumptions about the Programme, its context and its people. These assumptions were discussed and captured here.

## Abbreviations

AKU: Aga Khan University
AREF: Africa Research Excellence Fund
CAN: Crick Africa Network
CISM: Centro de Investigação em Saúde de Manhiça
GCRF: Global Challenges Research Fund
LIMS: Laboratory Information Management System
LSHTM: London School of Hygiene and Tropical Medicine
ODA: Official Development Assistance
PRECISE: PREgnancy Care Integrating translational Science, Everywhere
RECAP: Research capacity building and knowledge generation to support preparedness and response to humanitarian crises and epidemics
TGHN: The Global Health Network
UKRI: United Kingdom Research and Innovation
